# Optimization of robustness of interdependent network controllability by redundant design

**DOI:** 10.1371/journal.pone.0192874

**Published:** 2018-02-13

**Authors:** Zenghu Zhang, Yongfeng Yin, Xin Zhang, Lijun Liu

**Affiliations:** School of Reliability and System Engineering, Beihang University, Beijing, China; Universidad Nacional de Mar del Plata, ARGENTINA

## Abstract

Controllability of complex networks has been a hot topic in recent years. Real networks regarded as interdependent networks are always coupled together by multiple networks. The cascading process of interdependent networks including interdependent failure and overload failure will destroy the robustness of controllability for the whole network. Therefore, the optimization of the robustness of interdependent network controllability is of great importance in the research area of complex networks. In this paper, based on the model of interdependent networks constructed first, we determine the cascading process under different proportions of node attacks. Then, the structural controllability of interdependent networks is measured by the minimum driver nodes. Furthermore, we propose a parameter which can be obtained by the structure and minimum driver set of interdependent networks under different proportions of node attacks and analyze the robustness for interdependent network controllability. Finally, we optimize the robustness of interdependent network controllability by redundant design including node backup and redundancy edge backup and improve the redundant design by proposing different strategies according to their cost. Comparative strategies of redundant design are conducted to find the best strategy. Results shows that node backup and redundancy edge backup can indeed decrease those nodes suffering from failure and improve the robustness of controllability. Considering the cost of redundant design, we should choose BBS (betweenness-based strategy) or DBS (degree based strategy) for node backup and HDF(high degree first) for redundancy edge backup. Above all, our proposed strategies are feasible and effective at improving the robustness of interdependent network controllability.

## Introduction

With the increasingly wide and deep research into complex networks, such as traffic networks, energy networks, power networks and social networks, complex networks have drawn more and more attention in recent years. The control of complex networks is the focus and the ultimate purpose of studying them. Controlling complex networks means to make the networks reach the desired state by appropriate signal inputs. The first question is to ensure whether the complex networks are controllable or not.

Real systems are coupled by multiple networks and thus construct interdependent networks [1–4]. For example, the communication networks describe the networks where the vertex is one person in real world and the edge is one kind of communication between two persons. In fact, the communication networks are composed of online application network, email network and telephone network. As another example, the Internet network and the power network are mutually dependent. The failure of nodes in one network will lead to the failure of nodes that depend on the former in the other network. In fact, less research focuses on the controllability of interdependent networks.

Because of interactions of nodes in interdependent networks, the failure of a small fraction of nodes will cause the crash of the whole system. The network will then be out of control. Therefore, the robustness of interdependent network controllability is another topic that should be studied. This paper will analyse the robustness of interdependent network controllability and find methods to optimaze it.

A significant shortcoming of solving the controllability for complex networks by classical control theory is that it is a computationally prohibitive job for large scale networks, resulting from the need for brute-force search of all combinations of the rank of *W* [5, 6]. To bypass the requirement of measuring all combinations, Liu et al. proposed an analysis framework for the structural controllability of a single network [7]. As long as the network is structurally controllable, it will always be controllable by adjusting appropriate parameters of the network [8]. However, this framework can only solve the controllability of a single network without considering multiple networks.

With the development of the research in the field of controllability for complex network, a general question is what type of role each node plays. Based on the framework of controllability, Menichetti et al. [9] discussed the determined effect of nodes with minimal in-degree and out-degree on the controllability of the network. By comparing the controllability between an ER network and the maximum entropy network, Hou et al. found that the latter had a higher controllability [10]. This paper determined that the controllability of one node is influenced by the properties of its neighbors. Jia and Barabasi [11] put forward the concept of control capacity for nodes by calculating the frequency of a single node in all minimum matching sets. The author described a method that could be used to analyze the relationship between the control capacity and the degree of the nodes.

To measure the importance of nodes in the driver node set, Jia et al. [12] considered the classification of driver nodes based on the structural controllability of a complex network. This classification can successfully determine the role of one node on the controllability of a single network. However, although useful for the controllability of a single network, the research above has not considered the robustness of controllability for networks.

The model of cascading failure in a random network that includes only overload failures was presented by Watts DJ [13]. In this model, the load of a failed node will be reallocated to other functional nodes. Buldyrev et al. [14] studied the interdependent failure process in interdependent networks without considering overload failures. The failure of one node in one network will lead to the failure of a node in another network. Therefore, a small fraction of failures of nodes will crash the whole network. We will consider a cascading process including both the overload and the interdependent failures in our work.

Cohen et al. [15] was the first to consider theoretically failures in the structure via percolation theory. Most of the research on the robustness of complex networks focused on the structure of the network without considering controllability [14, 16–19]. To measure the robustness of controllability and global connectivity for a single network, a parameter was presented based on the maximum weakly connective components [20]. The result for the robustness of controllability based on edge attack shows the accuracy and usability of this parameter [21], giving an instructive idea for obtaining the parameter to evaluate the robustness of the interdependent network controllability.

Liu et al. [22] proposed a method of redundant design to optimize the structure of interdependent networks. To achieve the terminal purpose of optimizing the robustness of interdependent network controllability, we improved the redundant design by analyzing the factors which determine the process of cascading failures and proposing different strategies.

Based on the previous research results, this paper solves the optimization question of robustness for interdependent network controllability as the following idea: first, we construct the model of the interdependent networks. Second, we determine the process of cascading process including both overload and interdependent failures in interdependent networks under different proportions of node attacks. Then, we analyze the robustness of interdependent network controllability. Finally, a redundancy design and sevaral strategies are proposed to optimize the robustness of interdependent network controllability.

## Materials and methods

### Structural controllability of networks

In an N-dimensional space, the state of the nodes in a system can be described by an N-dimensional vector that is *x*(*t*) = (*x*_1_(*t*), *x*_2_(*t*), ⋯, *x*_*N*_(*t*))^*T*^. The system is controllable if it can reach any expected state from any initial state within a finite time [23].

For a nonlinear system, many aspects act similarly to time-invariant dynamics:

$$\begin{array}{c} \dot{x}\left(t\right)=Ax\left(t\right)+Bu\left(t\right), \end{array}$$ (1)

where *x*(*t*) = (*x*_1_(*t*), *x*_2_(*t*), ⋯, *x*_*N*_(*t*))^*T*^ denotes the state of the nodes at time *t*, $A={\left[\begin{array}{c} a_{ik} \end{array}\right]}_{N\times N}$ is the adjacency matrix of *N* nodes, $B={\left[\begin{array}{c} b_{ik} \end{array}\right]}_{N\times M}$ is the input matrix that identifies nodes driven by input signals, and *u*(*t*) = (*u*_1_(*t*), *u*_2_(*t*), ⋯, *u*_*N*_(*t*))^*T*^ is the vector of input signals.

Kalman controllability rank condition [23] says that the system expressed by Eq (1) is controllable if and only if the controllability matrix satisfies as follows:

$$\begin{array}{c} \text{rank}(W)=N, \end{array}$$ (2)

where *W* = [*B*, *AB*, ⋯, *A*^*N*-1^*B*].

To overcome the shortcomings of computationally prohibitive tasks to calculate Eq (2) for large networks, the notion of structural controllability [8] is proposed. The system described by Eq (1) is structurally controllable if the non-zero weights in *A* and *B* can be replaced by some parameters so that the system satisfies Eq (2). A system that is structurally controllable can be controlled in most circumstances [8]. The least number of input signals that can be used to control a network is denoted by *N*_*D*_. Therefore, *N*_*D*_ can describe the controllability of the network, where a higher *N*_*D*_ means more input signals to control the network. Liu et al. [7] proposed a controllability analysis framework for a complex network based on the maximum matching theory to solve the minimum set of driver nodes that are the unmatched nodes of the maximum matching set in the directed network.

**Fig 1:**
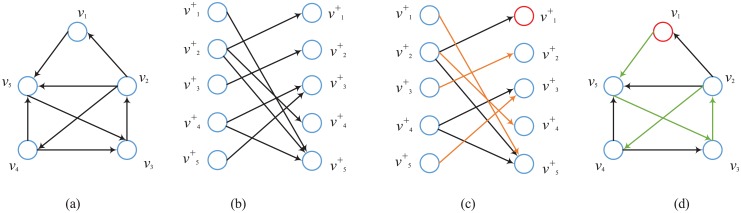
Demonstration of calculating the minimum set of driver nodes. (a) The directed network topology with five nodes. (b) The bipartite network of the directed network. Each connection in the bipartite network is related to an edge in the directed network. (c) The max matching set of the bipartite network. The set of red edges is the max matching set where any additional edge will make some vertex matched more than one time. The red vertex is the minimum set of driver nodes. (d) The max matching and minimum set of driver nodes in the directed network.The red vertex is the minimum set of driver nodes.The set of green edges is the max matching set in the directed network.

The undirected network can be regarded as a directed network where there are two edges between any nodes. Moreover, the directed network can also be regarded as a bipartite network. In the bipartite network, $H\left(A\right)=(V_{A}^{+},V_{A}^{-},\Gamma )$, where $V_{A}^{+}=\{x_{1}^{+},\cdots ,x_{N}^{+}\}$ and $V_{A}^{-}=\{x_{1}^{-},\cdots ,x_{N}^{-}\}$ denote the node sets in the complex network and $\Gamma =\{(x_{j}^{+},x_{i}^{-})|a_{ij}\neq 0\}$ denotes the set of edges. Fig 1 shows the bipartite network of a directed network *G*(*A*) and the process to calculate the minimum set of driver nodes. The minimum set of driver nodes can be obtained by solving the maximum matching of the bipartite networks where the unmatched nodes are exactly the minimum set of driver nodes to make the network structurally controllable.

Each node in the complex network can be classified into three different types according to its role in maintaining controllability [7]: (1) critical if it will always exist in all minimum sets of driver nodes; (2) redundant if will never exist in any minimum set of driver nodes; and (3) ordinary if it is neither critical nor redundant.

### Cascading process of interdependent networks

Suppose that a single network *G* is composed of a vertex set *V* and an edge set *E*, where *V* is {*v*_1_, *v*_2_, ⋯, *v*_*N*_} and *E* is {*e*_1_, *e*_2_, ⋯, *e*_*N*_}. A path between node *v*_*i*_ and node *v*_*j*_ is a subset of consecutive edges that can be expressed as *P*(*v*_*i*_, *v*_*j*_). So |*P*(*v*_*i*_, *v*_*j*_)| is the length of path *P*(*v*_*i*_, *v*_*j*_). Moreover, *d*_*ij*_ is defined as the shortest length between node *v*_*i*_ and node *v*_*j*_. There may exist several paths *P*(*v*_*i*_, *v*_*j*_) whose length is equal to *d*_*ij*_.

According to the topology of a complex network, network failures on a global scale can be caused by local node failures through the cascading mechanism. The initial load on each node of a complex network can be denoted by its betweenness centrality, which means the total number of the shortest paths passing through it. Its formula is expressed as follows:

$$\begin{array}{c} B^{0}\left(v_{k}\right)=\sum_{i\neq j=1}^{N}\frac{N_{ij}\left(k\right)}{N_{ij}} \end{array}$$ (3)

where *N*_*ij*_(*k*) denotes the number of the shortest paths *P*(*v*_*i*_, *v*_*j*_) that passes through node *v*_*k*_, and *N*_*ij*_ means the number of the shortest paths between node *v*_*i*_ and node *v*_*j*_.

Because the capacity of a node is limited, the loads of nodes in the complex network will be reallocated and exceed their limits as a result of attacks on the complex network. Kim and Motter [24] found that there is a nonlinear relationship between the load of a node and capacity. Dou B. [25] proposed a nonlinear load-capacity model for real systems that can be expressed as follows:

$$\begin{array}{c} Cap\left(v_{k}\right)=B^{0}\left(v_{k}\right)+\beta {\left(B^{0}\left(v_{k}\right)\right)}^{\alpha },k=1,2,\cdots ,N \end{array}$$ (4)

where *α* > 0, *β* > 0. When *α* = 1, this model degenerates to linear load-capacity model. Suppose that the load of a failed node will be assigned to other functional nodes on average which can be expressed as follows:

$$\begin{array}{c} B^{t}\left(v_{j}\right)=B^{t-1}\left(v_{j}\right)+\frac{\sum B^{t-1}\left(v_{i}\right)}{|V_{functional}|},v_{i}\in V_{failed},v_{j}\in V_{functional} \end{array}$$ (5)

where *V*_functional_ and *V*_failed_ are sets of nodes that remain functional and failed, respectively. |*V*_functional_| denotes the number of nodes in *V*_functional_. If the load of a node is larger than its capacity, this node will cause overload failure. Moreover, to measure the cost of a network, we define the cost of the network according to the capacities of the nodes. The greater the value of *α* and *β*, the more resources that should be allocated on the network. The cost of a network is defined as follows:

$$\begin{array}{c} \text{Cost}=\sum_{i=1}^{N}Cap(v_{i}) \end{array}$$ (6)

Real systems are always coupled together by multiple networks and should be regarded as interdependent networks. For example, the power network and the Internet network are coupled together so that the power stations rely on Internet nodes for control and vice versa. The significant feature of interdependent networks is that the failure of nodes in one network will lead to interdependent failures of nodes in the other network [14]. Removal of a small fraction of nodes in one network will cause a considerable fraction of interdependent failures in the whole network.

This paper considers both the overload failures and the interdependent failures in the cascading process of interdependent networks. We begin by removing a fraction of nodes in one network and all the edges connected to the removed nodes. Then, the process of interdependent failures happens, which leads to the removal of all edges that are connected to different clusters in the other network, and removal of edges and isolated nodes in the other network will cause further interdependent failures until no nodes fail. As a result of removing nodes in the network, the load of failure nodes will be reallocated to those remaining nodes. Once the load of a node exceeds its capacity and fails, the process of overload failures will occur. This is another failure mode of nodes in the interdependent networks which in turn causes further interdependent failures of the network.

**Fig 2:**
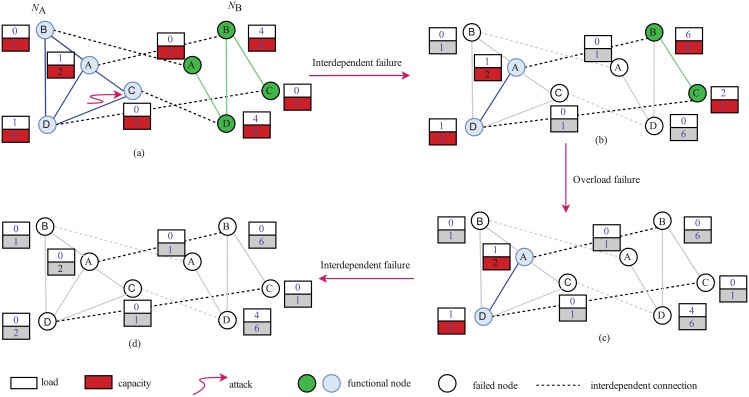
Cascading process of interdependent networks under node attacks. (a) The topology of interdependent networks with eight nodes. The initial attack set of nodes is *C*. (b) The topology of the networks after the process of interdependent failure. Nodes *A*, *D* in network *N*_*B*_ and nodes *B*, *C* in network *N*_*A*_ fail and lose their functions. (c) The topology of the networks after the process of overload failure. Node *C* in network *N*_*B*_ fails as the load exceeds its capacity. So is node *B*. (d) The terminal state of the interdependent networks. All nodes fail and lose their functions.

Fig 2 illustrates the process of cascading failures under node attacks in interdependent networks. We consider for simplicity, and without loss of generality, two networks, *N*_*A*_ and *N*_*B*_, with the same number of four nodes as depicted in Fig 2(a). The black dashed lines that cannot transfer traffic are dependency edges between two isolated networks. However, the blue and green lines in two networks that can transfer traffic are connective edges. At the first stage, node *C* in network *N*_*A*_ is attacked and fails. After the process of interdependent failures, nodes *A*, *D* in network *N*_*B*_ and nodes *B*, *C* in network *N*_*A*_ will fail and lose their functions. Then, their loads will be reassigned to nodes that are still functional in their own network, as shown in Fig 2(b). In the next stage, the load of node *C* in network *N*_*B*_ exceeds its capacity and fails. Then, node *D* fails in the same way as *C*. This is the process of overload failures of the networks. The result after overload failures can be seen in Fig 2(c). Then, another process of interdependent failures occurs. Nodes *A*, *D* in network *N*_*A*_ fail. Finally, all nodes fail and lose their functions as shown in Fig 2(d).

### Robustness of interdependent network controllability

The robustness of a complex network has become a hot topic in recent years [15, 21, 25, 26]. Previous work has mainly focused on the connectivity of complex networks. Some topological properties of the network will change after some nodes are attacked and fail. These properties are parameters for evaluating the robustness of the complex networks, such as the connectivity, the largest connected component, and the average shortest path. With the development of research on the controllability of complex networks, more attention is attracted by the robustness of the controllability of a complex network.

When a different fraction of nodes in interdependent networks is attacked, the network will reach a stable state in the end. Then, we analyze the controllability of the interdependent networks under different proportions of attacks to obtain the robustness of controllability in the interdependent networks. The parameter to evaluate the robustness of controllability in the interdependent networks can be calculated as follows:

$$\begin{array}{c} CR=\frac{1}{\left|N_{A}|+|N_{B}\right|}\sum_{q=1/N_{A}}^{1}\frac{s_{A}\left(q\right)+s_{B}\left(q\right)}{N_{D}^{A}\left(q\right)+N_{D}^{B}\left(q\right)}, \end{array}$$ (7)

where |*N*_*A*_|, |*N*_*B*_| are the number of nodes in networks *N*_*A*_ and *N*_*B*_, respectively. A directed network is called weakly connected if replacing all of its directed edges with undirected edges produces a connected (undirected) network. *s*_*A*_(*q*) is the fraction of the largest weakly connected component nodes of network *N*_*A*_ in the whole network after removing *qN*_*A*_ nodes in network *N*_*A*_ and *qN*_*B*_ nodes in network *N*_*B*_, and *s*_*B*_(*q*) is the fraction of the largest weakly connected component nodes of network *N*_*B*_ in the whole network. $N_{D}^{A}\left(q\right)$ is the number of minimum driver nodes of network *N*_*A*_ after removing nodes. $N_{D}^{B}\left(q\right)$ is the number of minimum driver nodes of network *N*_*B*_.

### Redundant design in interdependent networks

The more important purpose of studying the robustness of controllability of interdependent networks is to find methods to improve it [27, 28]. As the properties of interdependent networks are determined by the topology of each isolated network and the dependency edges between the networks, the robustness of controllability can be improved by node backup for each isolated network and dependency edge backup between each isolated network. This is a redundancy design of the interdependent networks [22].

#### Node backup

The overload failures exist in the cascading process of interdependent networks. When a node is attacked, its failure will lead to a higher load of the functional nodes in the network and cause overload failure. If nodes are backed up, they can stand more loads and enlarge their capacity. Therefore, node backup will suppress the process of overload failures and decrease the proportion of failure nodes. Furthermore, fewer minimum driver nodes to maintain the networks structurally controllable are required. At last, we can improve the robustness of controllability for interdependent networks.

**Fig 3:**
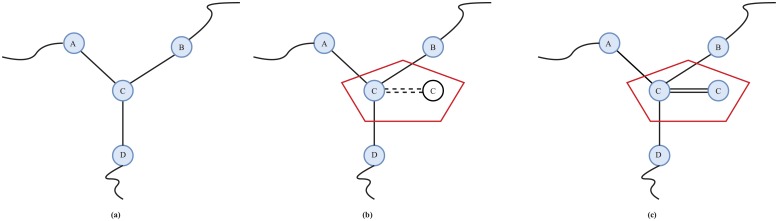
Model of node backup. (a) The topology of networks without node backup. (b) The node backup will not be activated if nodes *C* works. (c) The node backup will be activated if nodes *C* fails.

Fig 3 shows the model for node backup. Fig 3(a) is the topology of the nodes without backup. This paper assumes that the backup nodes will not be activated until necessary. Therefore, the initial loads of the nodes will not increase as shown in Fig 3(b), but the capacities of the nodes will increase. When node *C* fails, its load will allocate to its functional neighbors, which may cause overload failures. If we back up node *C* and its backup nodes has been activated, the overload failures will not happen as shown in Fig 3(c). Furthermore, the cost of the node *C* will also increase because of node backup. Clearly, the capacity of node *v*_*k*_ can be calculated as follows [22]:

$$\begin{array}{c} CC(v_{k})=n\times Cap(v_{k}),n\in N,k=1,\cdots ,N, \end{array}$$ (8)

where *Cap* (*v*_*k*_) is the initial capacity of node *v*_*k*_, and *n* is the number of backup nodes for node *v*_*k*_. The cost of the network after node backup is $\sum_{i=1}^{N}CC(v_{k})$.

The strategy to determine the set of backup nodes has a significant effect on the optimization of robustness of interdependent network controllability. This paper compares five different strategies to backup nodes in interdependent networks, which are random-based (RBS), low frequency of overload failures first (LFOF), high frequency of overload failures first (HFOF), degree based (DBS) and betweenness-based (BBS).

RBS strategy randomly selects nodes of interdependent networks to backup nodes. DBS strategy and BBS strategy select nodes that have a higher degree and a higher betweenness, respectively.

LFOF strategy prefers to select nodes that have a low frequency of overload failures that can be obtained by large scale simulation of cascading failures over in interdependent networks. In fact, the frequency data of overload failure nodes shall be obtained before we choose a strategy to select nodes to backup. The structure of the interdependent networks which produce the frequency data is the same as the structure of networks which will have redundancy design. To obtain the frequency data of failure nodes including overload failures and interdependent failures, attack proportion and enough simulations shall be choose appropriately as shown in S1 Mat and S2 Mat. Similarly, HFOF strategy prefers to select nodes that have a higher frequency of overload failures.

#### Dependency edge backup

**Fig 4:**
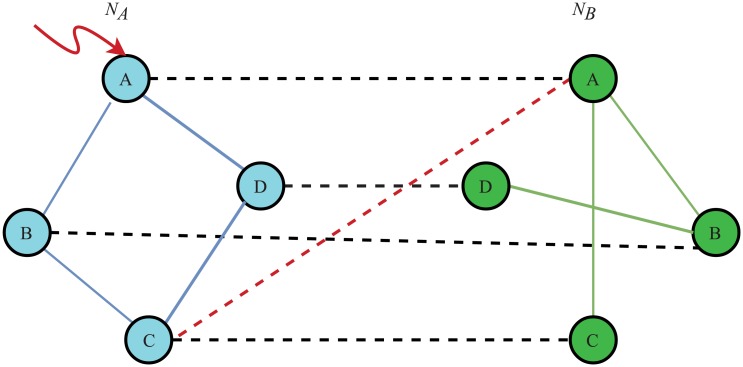
Dependency edge backup. If node *A* is attacked, node *A* in another network will not fail because of its dependency edge backup strategy.

Interdependent failure is another failure mode in the cascading process of interdependent networks. The removal of a node in network *N*_*A*_ will fail the node that depends on it in network *N*_*B*_. Fig 4 shows the mode of dependency edges backup. Node *A* in network *N*_*A*_ is attacked and node *A* in network *N*_*B*_ will fail if no dependency edges backup exists. However, if node *A* in network *N*_*B*_ has a dependency edge, as the red dashed line shows, it will activate this edge and depend on node *C* in network *N*_*A*_. Therefore, node *A* in network *N*_*B*_ will remain working. This redundancy design will suppress the interdependent failure process and increase the robustness of interdependent network controllability.

The dependency edge backup will not increase the initial loads and capacities of nodes in the network. However, some additional resources will be added to the network. Therefore, the cost of the dependency edges can be calculated as follows [22]:

$$\begin{array}{c} Cost\left(l_{ij}\right)=\max\left\{Cap\left(v_{i}\right),Cap\left(v_{j}\right)\right\},v_{i}\in V\left(N_{A}\right),v_{j}\in V\left(N_{B}\right) \end{array}$$ (9)

where *Cost*(*l*_*ij*_) is the cost of dependency edge *l*_*ij*_. The total cost of dependency edges is ∑*Cost*(*l*_*ij*_).

The strategy to determine those nodes that have dependency edges plays a key role in the result of optimizing the robustness of controllability. This paper has studied seven strategies, which are random selected (RSB), low frequency of interdependent failures first (LFIF), high frequency of interdependent failures first (HFIF), across low and high frequency of interdependent failures (AFIF), high degree first (HDF), low degree first (LDF) and across high and low degree (ADF).

RSB strategy randomly selects nodes and makes redundancy edges between them. LFIF strategy prefers to select a lower frequency of interdependent failure nodes in both networks and backup their redundancy edges. Similarity, HFIF prefers to select a higher frequency of interdependent failure nodes. AFIF selects one node with a higher frequency of interdependent failures in a network and a lower node in another network. HDF and LDF selects nodes with high and low degree in both networks, respectively. ADF strategy select a higher degree node in a network and a lower degree node in another.

### The procedure of the optimization algorithm for robustness

Redundancy design is a method to improve the robustness of controllability of interdependent networks. The process of the method is described as follows:

Step 1 Initiate the interdependent networks.
  In this stage, the topology of the interdependent networks will be initialized, including the scale of networks, the degree distribution of the network and the dependency edges between the networks. Therefore, we can get the initial loads and capacities of nodes in interdependent networks. One of the redundancy design strategies is also determined to optimize the robustness of controllability. According to the strategy, some changes will be made in the topology of networks.
Step 2 Obtain the frequency data of failure nodes including overload failures and interdependent failures in large scales simulation in interdependent networks.
  In this stage, the attack proportion shall be determined in order to produce sufficient frequency data of failure nodes. Then we randomly select the set of nodes in both networks to attack. Nodes which are attacked will fail and so will the nodes which depend on the former. This is the process of interdependent failures. The load of failure nodes will be reallocated to the nodes that are still functional. Once the load of a node exceeds the capacity of that node, the node will fail. Therefore, the process of overload failures will begin. Interdependent failures alternate with overload failures until no nodes fail. Then we obtain the frequency data of failure nodes including overload failures and interdependent failures by repeating this simulation for enough times.
Step 3 The cascading process of interdependent networks under different proportions of attack nodes.
  Firstly, we initialize the parameters of interdepenedent networks by step 1. The proportion of attack nodes is continuously obtained from the interval [0, 1]. We randomly select the set of nodes in both networks to attack. The cascading process, including the interdependent failures and overload failures, will alternately happen until the networks reach a stable state. Then, we calculate the minimum driver node set of the networks that are left and collect data in the cascading process such as the proportion of interdependent failures and overload proportion, the proportion of minimum driver nodes in both interdependent networks and the cost of the strategy. Repeated simulations are conducted under diffrent attack proportions. At last, we can obtain the robustness of interdependent network controllability by Eq (7) under different strategies from these data.
Step 4 Compare the effect of different strategies on optimization of the robustness of interdependent network controllability.

## Results

To verify the feasibility and effect of our proposed strategy on the optimization of the robustness of interdependent network controllability, we conduct a series of comparative experiments in this section. Without the loss of generality, the two isolated directed networks labeled *N*_*A*_ and *N*_*B*_ are ER networks that are widely used in the research of complex networks and have the same number of nodes that can be denoted by |*N*_*A*_| = |*N*_*B*_| = 300. The probabilities of two nodes that are connected are *p*_*A*_ = 0.02 and *p*_*B*_ = 0.02 in networks *N*_*A*_ and *N*_*B*_, respectively. This paper randomly selects initial dependency edges with one node in network *N*_*A*_ and and one in network *N*_*B*_. This is the initial topology of interdependent networks that is used in all simulations. For the load-capacity model, we use a nonlinear load-capacity model, where the parameter *α* is set to 0.97, and *β* is 6. For LFOF and HFOF strategies that are used to optimize the redundant design in node backup, we shall gather the frequency information of overload failures for each node in the interdependent networks. Similarity, HFIF, LFIF and AFIF strategies that can improve the performance of dependent edges backup require the frequency information of interdependent failures. To obtain the frequency data of the failure nodes, we simulate 100,000 times under the same conditions when the attacked proportion is 30%.

**Fig 5:**
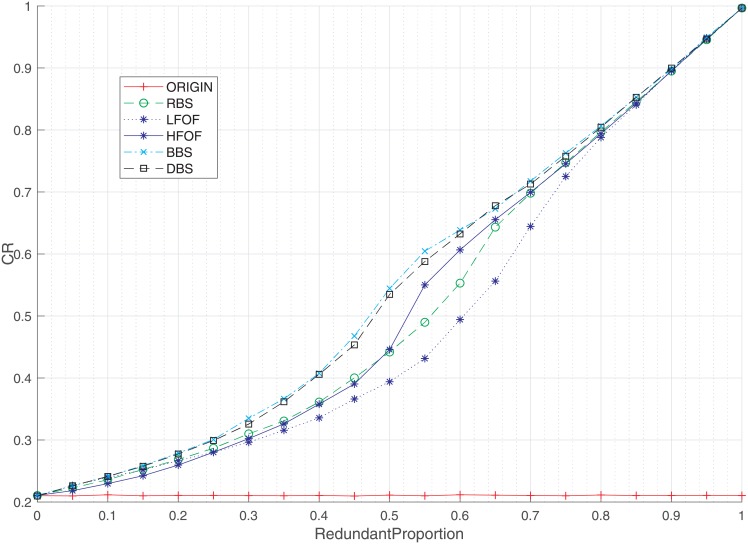
Relationship between the robustness of interdependent network controllability and the proportion of node backup under different backup strategies. CR is a parameter to evaluate the robustness of interdependent network controllability.

**Fig 6:**
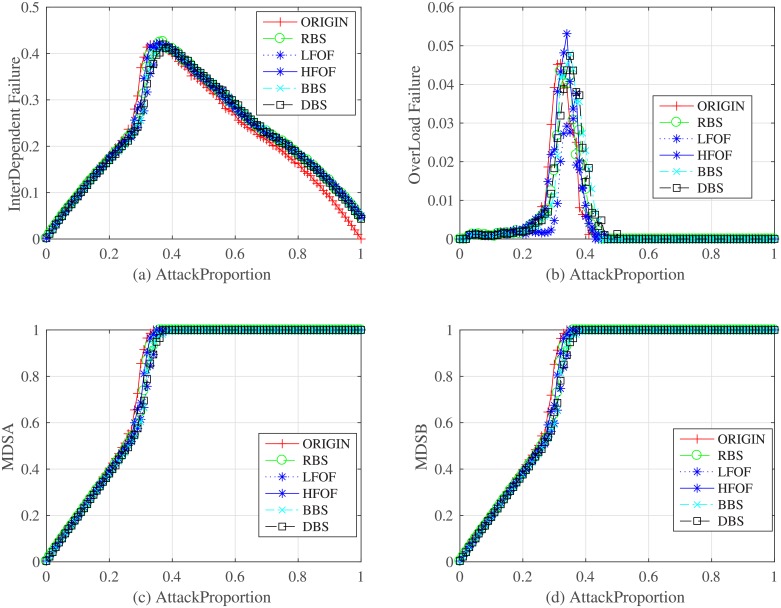
Statistical data for the network under the node backup proportion of 10% for different strategies.

**Fig 7:**
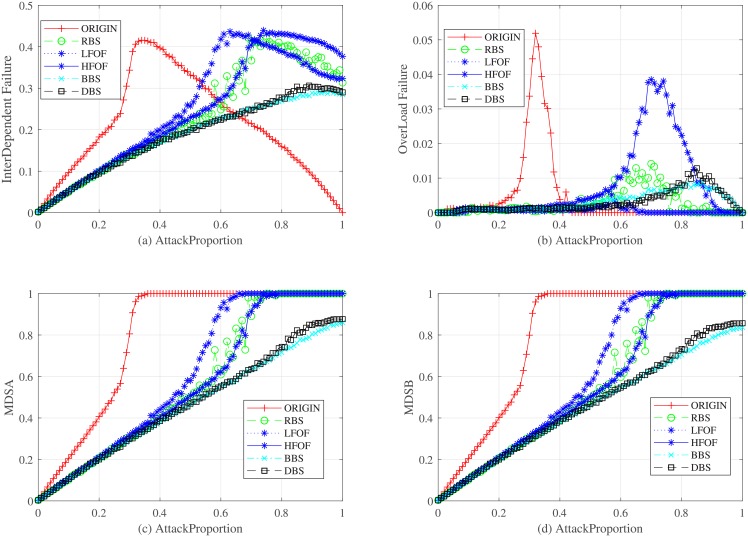
Statistical data for the network under the node backup proportion of 55% for different strategies.

**Fig 8:**
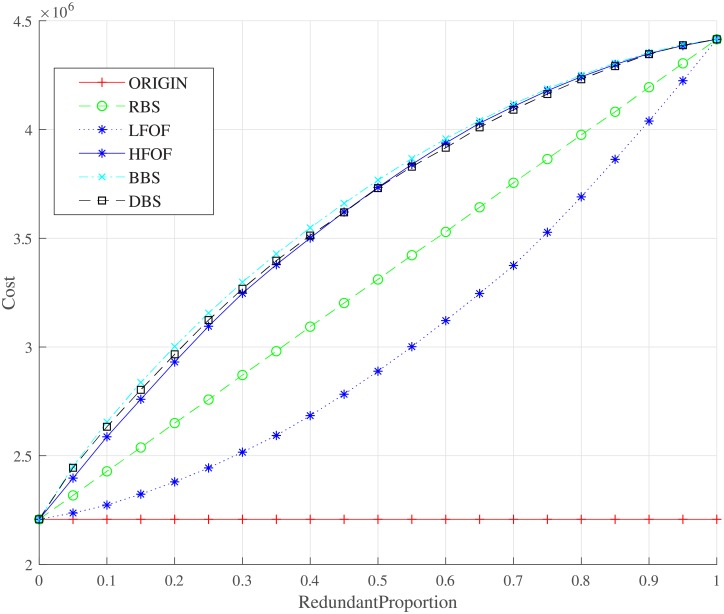
Relationship between the cost and the proportion of node backup under different backup strategies. The total cost of the interdependent networks with node backup can be obtained by Eq (8).

To compare the performance of strategies over the node backup mechanism, experiments based on the five strategies (RBS, LFOF, HFOF, BBS, and DBS) are conducted as depicted in Figs 5–8. The red line labeled as ORIGN in each figure shows the result without redundant design. For different proportions of node backup, the robustness of interdependent networks under different strategies is shown in Fig 5. The robustness of controllability increases slowly as the redundant proportion is small and speeds up when the redundant proportion increases, showing the feasible method of node backup to optimize the robustness. From another perspective, BBS and DBS strategies can make interdependent networks more roubust and work better than other strategies in the same condition. Therefore, nodes with higher degree and load urgently need backups. Furthermore, node backup for lower failure frequency (LFOF) is worse than node backup for higher nodes (HFOF) and even worse than the RBS strategy, demonstrating that we shall allocate more resources to nodes that failed more in the past. Therefore, LFOF strategy is a bad choice to optimize the robustness of interdependent networks.

Figs 6 and 7 shows statistical data including interdependent failures and overload failures for the network under the node backup proportions of 10% and 55% for different strategies. MSDA and MSDB are the proportions of driver nodes in network *N*_*A*_ and *N*_*B*_, respectly. We can ensure that the node backup strategy can indeed decrease those nodes suffering from failure and improve the robustness of controllability. It will delay the appearance of a phase transition behavior as the attack proportion grows. The cost of different strategies is described in Fig 8 where we can observe that DBS and BBS will have a high cost though they can improve the robustness of controllability at the same time. We must take care that the HFOF strategy costs nearly the same as the DBS and the BBS strategies. Under these conditions, HFOF is not a good strategy, nor is LFOF. Therefore, we should choose the BBS or DBS strategy for node backup.

**Fig 9:**
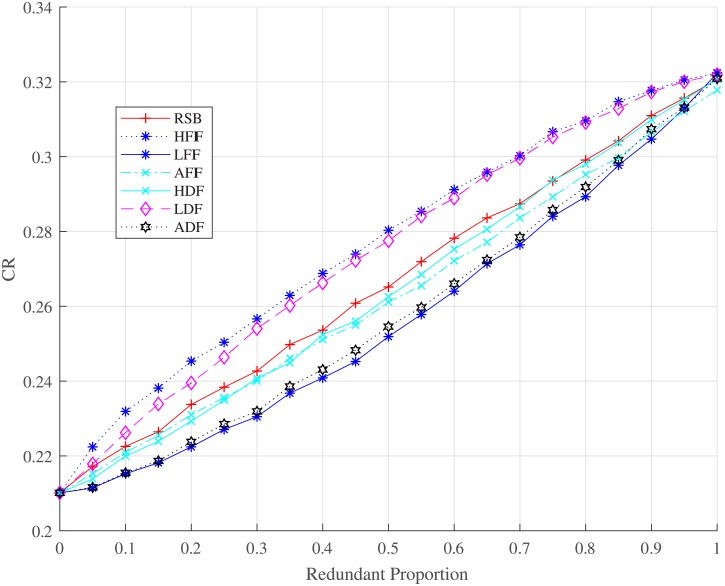
Relationship between the robustness of interdependent network controllability and the proportion of redundancy edge backup under different backup strategies. CR is a parameter to evaluate the robustness of interdependent network controllability.

**Fig 10:**
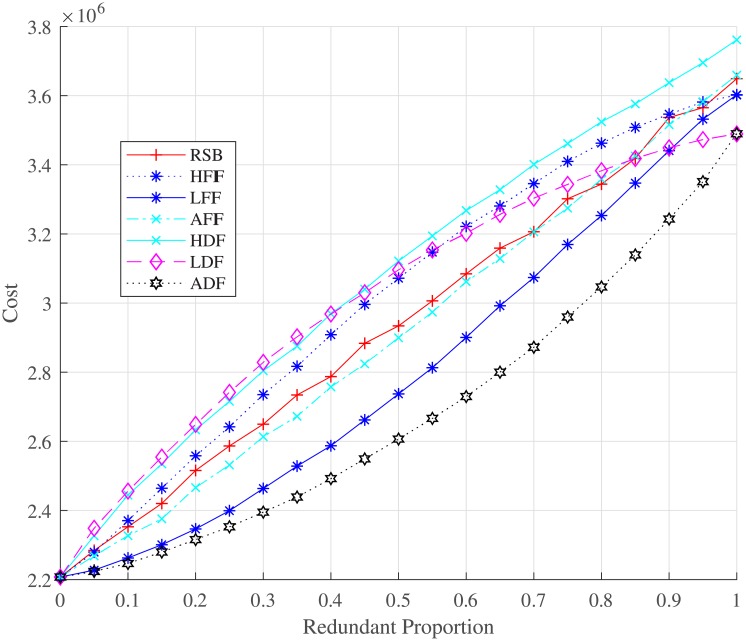
Relationship between the cost and the proportion of redundancy edge backup under different backup strategies. The total cost of the interdependent networks with redundancy edge backup can be obtained by Eq (9).

By comparing the performance of strategies over the redundancy edges backup mechanism, we carry out our simulation based on the seven strategies (RSB, LFIF, HFIF, AFIF, ADF, HDF, and LDF) as shown in Figs 9 and 10. For different redundant proportions, Fig 9 presents the relationship between the robustness of controllability and redundancy edges backup strategies. The HFIF and HDF strategies work better than the others. Therefore, we shall choose redundancy edges that have nodes with higher degree or higher failure frequency. LDF and LFIF are not good choices to optimize the robustness of controllability. They work even worse than the RSB strategy. The cost of all strategies for redundancy edges backup is demonstrated in Fig 10. We can see that HDF costs more than HFIF when the redundant proportion ranges from 0 to 0.55. However, HDF will cost less when the redundant proportion is larger than 0.55. The robustness of all redundancy edge backup strategies converges to 0.32 when redundant proportion is close to 1 because redundancy edges backup cannot suppress the process of overload failure, which can fail the isolated network. Therefore, dependency edge backup can only optimize the interdependent failure process and improve partial robustness but not all robustness.

## Discussion

Controllability of networks has become hot topic in recent years. The cascading process of interdependent networks, including interdependent failure and overload failure, could make a whole network cascade when only a small fraction of the nodes fail in fault. In this paper, we have studied the cascading process based on the non-linear load-capacity model. We analyze the factors which determine the process of cascading failures and proposing a redundant design including node backup and dependency edge backup to optimize the robustness of interdependent network controllability. The redundant design is feasible and effective at improving the robustness of interdependent networks. As the strategy to determine the set of backup nodes and denpendency edges has a significant effect on the optimization of robustness of interdependent network controllability, this paper compares five node backup strategies and seven dependency edge backup strategies in interdependent networks. We also considered the cost of all the strategies.

Among the strategies which are proposed in this paper, the BBS and DBS strategies are good choices for node backup, even though they cost more than the other strategies. Similarity, the HFIF and HDF strategies are best choices of redundancy edges backup. Considering the cost of the strategy, HDF is better than HFIF when the redundant proportion of redundancy edges is small, and HFIF is the best when the redundant proportion of redundancy edges is large.

## Supporting information

S1 Fig The structure of interdependent networks without redundant design under different attack proportions. The parameters of the ER interdependent networks is *α* = 0.97, *β* = 2, 6, 10, *N* = 300, *p*_*a*_ = 0.02, *p*_*b*_ = 0.03, MDSA is the number of minimum driver nodes in network *N*_*A*_, so is MSDB.
(FIG)

S2 Fig The structure of interdependent networks for different *α* when attack proportions is 5%. The parameters of the ER interdependent networks is *β* = 2, 6, 10, *N* = 300, *p*_*a*_ = 0.02, *p*_*b*_ = 0.03.
(FIG)

S1 Mat The structure of the interdependent networks. The parameters of the ER interdependent networks is *N* = 300, *p*_*a*_ = 0.02, *p*_*b*_ = 0.03.
(MAT)

S2 Mat The failure frequency data of nodes after simulation for 100000 times when the attack proportion is 30%. The parameters of the ER interdependent networks is *β* = 6, *α* = 0.97, *N* = 300, *p*_*a*_ = 0.02, *p*_*b*_ = 0.03. Sufficient frequency data of failure nodes can be obtained under the attack proportion.
(MAT)
